# The effect of community based health insurance on catastrophic health expenditure in Northeast Ethiopia: A cross sectional study

**DOI:** 10.1371/journal.pone.0205972

**Published:** 2018-10-18

**Authors:** Asnakew Molla Mekonen, Measho Gebreslassie Gebregziabher, Alemayehu Shimeka Teferra

**Affiliations:** 1 Department of Health Service Management and Health Economics, Institute of Public Health, College of Medicine and Health Sciences, University of Gondar, Gondar, Ethiopia; 2 Department of Health System, School of Public Health, College of Health Sciences, Mekelle University, Mekelle, Ethiopia; 3 Department of Epidemiology and Biostatistics, Institute of Public Health, College of Medicine and Health Sciences, University of Gondar, Gondar, Ethiopia; University of Liverpool, UNITED KINGDOM

## Abstract

**Introduction:**

Moving towards the goal of universal health coverage requires strengthening service delivery and overcoming significant financial barriers. The Government of Ethiopia is rolling out community based health insurance to protect the rural community from high out of pocket health expenditure and improve health service utilization. We investigated the effect of community based health insurance on catastrophic health expenditure in Northeast Ethiopia.

**Methods:**

A community based cross sectional study was conducted. A Multi stage sampling technique was used to get a total of 454 (224 insured and 230 uninsured) households. The data were entered using EPI info version 7 and analyzed using SPSS version 20 and STATA version 13 for binary logistic regression analysis and propensity score matching analysis respectively. Wealth status of the households was computed by Principal Component Analysis (PCA). A multivariable logistic regression analysis was done to identify the predictors of catastrophic health expenditure. Propensity score matching analysis was used to determine the effect of community based health insurance on catastrophic health expenditure. The average treatment effect on the treated (ATT) was calculated to compare the means of outcomes across insured and uninsured households.

**Results:**

A total of 454 household heads were included in the study, making a response rate of 91.2%.The total level of catastrophic health expenditure was found to be 20%. Among the households with catastrophic health expenditure, 4.41% were insured, whereas the remaining 15.64% were noninsured. Insured households (AOR = 0.19, 95% CI: 0.11–0.34), rich households (AOR = 1.98; 95% CI: 1.07–3.66), having member with chronic illness (AOR = 2.13, 95% CI: 1.01–4.51) and having member encountered any illness during the past 3 months (AOR = 2.44, 95% CI: 1.35–4.40) were statistically associated with catastrophic health expenditure. Community based health insurance contributed to 23.2% (*t* = -5.94) (95% CI: -0.31_-0.15) reduction of catastrophic health expenditure.

**Conclusion:**

The overall level of catastrophic health expenditure was high among noninsured households. Community based health insurance has significant financial protection from catastrophic health expenditure in northeast Ethiopia. Thus, the government need to scale up community based health insurance to protect the noninsured households from catastrophic health expenditure.

## Introduction

Moving towards the goal of universal health coverage will require strengthening service delivery and overcoming significant financial barriers. However, globally, every year around 150 million people suffer from financial catastrophe and about 100 million are pushed into poverty because of high out of pocket payments for health care services [1–3]. Majority of these people reside in developing countries [1–3]. Catastrophic health expenditure occurs when the out of pocket (OOP) payments for the health services consume such a large portion of the household’s available income and the household is pushed in to poverty as a result [3, 4]. Financial risk protection from high out-of-pocket payment (OOP) of the households and individuals can be achieved either by risk pooling through health insurance scheme or by funding the health services.

Despite there has been a significant improvement in health outcomes over the last two decades, Ethiopia is an underfinanced country when compared to sub-Saharan average. According to the fifth national health account, at an estimated $21 per capita, Ethiopia’s health spending remains one of the lowest in the world [5]. Out-of-pocket (OOP) payment at the time of seeking health care continues to be one of the major sources of financing for health in Ethiopia [5, 6]. Health care financing in Ethiopia is composed of donor financing, direct out-of-pocket (OOP) expenditure financing and the government financing respectively 49.9%, 33.7% and 15.6% in 2010/2011 [5–7]. Direct payment for seeking care is considered as regressive as it inhibits access to the health services for the poor. It is also considered to contribute to the impoverishment of families due to having to pay for unexpected health care services at the time of illness [8]. The availability of health services requiring payment, low capacity to pay for health care, and unavailability of health insurance are the commonly identified causes of catastrophic health expenditure ([8, 9].

The proportion of households facing catastrophic health expenditure from out-of-pocket (OOP) payments broadly varies between countries, that is, it ranges from less than 1% in some countries to greater than 10% in others [9]. Many countries have been implementing prepayment schemes for protecting the households’ catastrophic health spending [10,11].

Ethiopia, with the aim of achieving universal health coverage by substantially reducing out-of-pocket spending for health care and increasing utilization of health services, established community-based health insurance scheme in 2011[11,12]. As part of the new health sector financing reform initiatives, the Ethiopian government has first launched a pilot community based health insurance scheme in 13 districts across the four main regions of the country (Tigray, Amhara, Oromia and SNNP) in June 2011 and is now being scaled up to reach a target of 80% of people in 80% of woredas by 2020. The community component of the CBHI scheme partly emanates from the fact that it is the community at the Kebelle (the lowest administrative unit) level that determines based on a simple majority vote whether or not to join the scheme. Once a Kebelle accepts the CBHI, the household makes voluntary decision whether to enroll to the scheme or not. Enrolment to the scheme is at the level of the household level rather than the individual. [13] The community is also involved in scheme management and supervision. The benefit packages, registration fees and premiums are the same within regions but there is slight variation across regions. The premium payment methods also slightly differ across regions. In Amhara region, the unit of contribution differs by family size, occupation and residence (urban and rural) [14].

Kebelle level CBHI officials and community representatives have the mandate to adjust the interval of premium collection based on their local conditions. The scheme is subsidized by both the central, regional governments. The central government provides a general subsidy amounting to a quarter of the premium collected at district level while the regional level governments cover the costs of providing a fee waiver for the poorest 10 percent of the population.

The benefit package includes both outpatient and inpatient service utilization at public facilities. It also includes food services, drug, laboratory and imaging services. Enrolled households may not seek care in private facilities unless a particular service or drug is unavailable at the contracted public facility. The scheme excludes treatment abroad, transportation and treatments with large cosmetic value such as artificial teeth and plastic surgery. The scheme has a referral procedure: members are expected to first visit health centers and may only seek care at hospitals (district or regional) if they have referral letters from these facilities. Those who do not follow this referral procedure need to cover half the costs of their medical treatment [13, 14].

According to studies conducted elsewhere [15–17], insured households have a lower probability of reaching the financial catastrophic threshold. Similarly, a national pilot community-based health insurance evaluation showed that the incidence of out of pocket payment of the CBHI members was on average about half of the nonmembers accessing health care [12]. However, studies conducted in China revealed that the health insurance scheme has failed to prevent the households from catastrophic health expenditure (CHE) and medical impoverishment [18, 19]. Likewise, studies in India and Vietnam reported that community-based health insurance has no substantial effect on financial protection and did not significantly protect the community from catastrophic health expenditure [20, 21].

Little, however, is known about the effect of community health insurance on catastrophic health expenditure in Ethiopia. Thus, knowledge about the effect of community-based health insurance on catastrophic health expenditure and its determinants is vital for evidence based decision making in the implementation and scale up of the community- based health insurance in Ethiopia.

## Materials and methods

A community based cross sectional study was conducted from February 01–30, 2016 among 454 households in Tehuledere district, Amhara Regional State, Northeast Ethiopia. Tehuledere district is found 431 kilometers away from Addis Ababa, the capital city of Ethiopia. The district has 19 health posts, 5 health centers and one district hospital. According to 2016 population projection, the total rural population of the district is 105,987, of which 57,270 are males. There were around 24,649 rural households in the district. The district has 54 health extension workers who are working as front line health service providers. Tehuledere district was one of the three pilot areas of community-based health insurance in the Amhara National Regional State which was started in June 2011.

The study populations were randomly selected households found in the selected kebeles of Tehuledere district. Households’ with critically ill household head/spouse during the study period and new CBHI members (less than a year since enrollment) were excluded from the study. Sample size for this study was calculated using double population proportion formula. Where p1(proportion of catastrophic health expenditure on noninsured households) = 19.38% [12] and p2(proportion of catastrophic health expenditure on insured households) = 6.83% [12], 80% power, a two-sided type I error of 5% and 95% confidence level to detect difference between insured and noninsured households. A ratio of insured to uninsured was 1:1. The total sample was 454 (224 insured and 230 uninsured households) after accounting design effect of two and 10% non-response rate.

A multi-stage sampling was applied to select the study population. In the first stage, four kebeles were randomly selected by simple random sampling out of 19 rural kebeles of the district. Data from Tehuledere district CBHI coordinating office was used to select insured and noninsured households based on their family folder number. Then, systematic random sampling was applied to select the eligible households from each Kebelle proportionally. The first household was selected randomly from the list of households’ family folder numbers.

The outcome variable was catastrophic health expenditure whereas community based health insurance was the treatment variable. The predictor variables were; demographic variables (age, sex, marital status, religion of household head/spouse and family size), socio economic variables (wealth index, education and occupation), and health and health related variables (presence of chronic illness, acute illness, health status of household, under 5 children, and above 50 household members).

### Measuring catastrophic health expenditure

There is no universally accepted consensus in the existing literatures on the threshold proportion of household’s expenditure [22]. However, there is an agreement that catastrophic health expenditure is an out of pocket payment (OOP) expenditure that exceeds the annual threshold of household’s total consumption or non-food consumption [9, 23]. WHO proposes that health expenditure should be considered to be catastrophic whenever it is greater than or equal to 40% of the household’s capacity to pay (non-subsistence spending). However individual countries could adopt a lower or higher percentage according to their situation [4]. Unlike the high income countries, health expenditures over a threshold, as low as 10% of the income or consumption expenditure of the households, may lead to catastrophic consequences in low and middle countries [8, 9]. A national CBHI evaluation in Ethiopia used 15% and 25% of nonfood expenditure as a cut off point for the occurrence of catastrophic health expenditure in the households [12].For this study, catastrophic health expenditure (CHE) occurred when a household’s total out of pocket health payment is greater than or equals to15% of household’s nonfood expenditure (non-subsistence spending) which was adopted from the national CBHI evaluation [12].

Households with CHE_H_ if OOPhNFEh≥15%

Households without CHE_H_ if OOPhNFEh≤15%

Out of pocket (OOP) health payments were paid by households at the point where they received health services; such as, consultation fees, drugs, hospital bills, traditional medicine and transportation for health. Nonfood expenditure (NFE) was calculated by using the last 12 months consumption for clothes and related, housing and related, social obligations, health, education, agriculture inputs and livestock and death related expenditures. The burden of health payments are the out-of-pocket payments as a percentage of a household’s nonfood expenditure.

The questionnaire for this study were adopted from Ethiopian health insurance agency household survey questionnaire [12]. Structured interviewer administered questionnaire was prepared in English and then translated to Amharic by language expert. Then, it was retranslated back to English to check for conceptual equivalence. The data were collected by six health extension workers and the data collection process was supervised of two bachelor holder nurses. A two days training was given to the data collectors and the supervisors on the data collection process.

Pre-test was done at Kalu district on 10% of sample households and the questionnaire were revised accordingly. Frequent supervision was conducted by the supervisors and principal investigator to ensure the completeness and consistency of the gathered information.

### Data processing and analysis

The data were entered using EPI info version7 software and analyzed using SPSS version 20 and STATA version 13 statistical packages. SPSS was used for the binary logistic regression analysis whereas STATA was used for propensity score matching analysis. Wealth status of the households was computed by Principal Component Analysis (PCA). Data cleaning was performed to check for frequencies, accuracy, consistencies and missing values. Frequencies, proportion, and summary statistics were used to describe the study population in relation to the study variables. Bivariate and multivariable logistic regression was conducted to investigate the effect of each independent on the dependent variable. Multivariable logistic regression was used to control the effect of confounding. Independent variables with p value of less than 0.2 were taken in to the multivariable logistic regression analysis to identify the predictors of catastrophic health expenditure.

Propensity score matching analysis was used to estimate the effect of community based health insurance on catastrophic health expenditure. Kernel matching was used to match insured and noninsured households based on propensity scores. We applied this approach because it uses all data which maximizes information gain and it has been used in similar study [24]. The average CBHI on the insured (ATT) was calculated by averaging the difference between the catastrophic health expenditure (CHE) of the insured households and that of the noninsured households after matching using propensity score. A t-test between the outcomes for the insured and noninsured households and 95% CI were computed.

Outcome variable (catastrophic health expenditure), the treatment variable (community based health insurance status) and covariates (age of household head/spouse, family size, health status of household, members with chronic illness/disability, under5 children, above 50 years member, member with acute illness, wealth status) were used to calculate propensity score and ATT. Common support region was determined to ensure that households had a positive probability of being insured or noninsured. Goodness of fit of logistic model was tested by Hosmer-Lemeshow test. Standard error was computed through bootstrapping with 100 replications to adjust for the additional sources of variability introduced by the estimation of the propensity score and the matching process itself.

### Ethical considerations

Ethical clearance was obtained from Institutional Review Board of Institute of Public Health, College Medicine and Health Science, and University of Gondar with the reference number of IPH/2865/02/2016. Permission letter was obtained from Amhara Regional Health Bureau, South Wollo health office and the district health office of Tehuledere. Informed verbal consent was obtained from each study participants. We have used informed verbal consent because the study was not sensitive and it has not any harm or procedure on the study subjects. We didn’t record or document the respondents’ consent; rather applicable consent form and the information sheet were integrated along with the respective data collection instrument. The informed verbal consent was approved by the Institutional Review Board of Institute of Public Health, College Medicine and Health Science, and University of Gondar.

It was clearly stated that their response was only for research purpose and the collected data were kept confidential. Participation in the study was on a voluntary basis. Participants who were unwilling to participate in the study and those who wish to quit from the study at any point in time were informed to do so without any restriction.

## Results

### Demographic and socio economic characteristics of household head/spouse

From a total of 454 household heads were participated in the study. Two hundred twenty four were insured households. More than half of the respondents were male (68.10%). Almost all of the respondents were Muslims (96.70%). Majority of the households 373(82.16%) were farmers. More than three fourth (79.96%) of the respondents were currently married. The mean family size of the respondent was 4.2 (±1.6) [Table 1].

**Table 1:** Demographic and socio economic characteristics of the household head/spouse by CHE status, Tehuledere district, Amhara regional state, 2016 [n = 454].

Characteristics household head/spouse	Catastrophic health expenditure		Total (%)
	Yes (%)	No (%)	
Household head/spouse age			
20–29	10(10.99)	30(8.26)	40(8.81)
30–39	23(25.27)	100(27.55)	123(27.09)
40–49	22(24.18)	111(30.58)	133(29.30)
50–59	10(10.99)	59(16.25)	69(15.20)
> = 60	26(28.57)	63(17.36)	89(19.60)
Household head/spouse sex			
Male	56(61.52)	253(69.70)	309(68.06)
Female	35(38.46)	110(30.30)	145(31.94)
Household head marital status			
Single	5(5.49)	16(4.41)	21(4.63)
Married	66(72.53)	297(81.82)	363(79.96)
Divorced	6(6.59)	23(6.34)	29(6.39)
Widowed	14(15.39)	27(7.44)	41(9.03)
Household head/spouse religion			
Muslim	86(94.51)	353(97.25)	439(96.70)
Orthodox	5(5.49)	10(2.75)	15(3.30)
Size of the household			
= <5	67(73.63)	285(78.51)	352(77.53)
>5	24(26.37)	78(21.49)	102(22.47)
Educational status			
No formal education	68(74.73)	256(70.52)	324(71.37)
Primary and above education	23(25.27)	107(29.48)	130(28.63)
Occupation/employment			
Farming	68(74.73)	305(84.02)	373(82.16)
Non farming	23(25.27)	58(15.98)	81(17.84)
Wealth status			
Poor	24(26.37)	127(34.99)	151(33.26)
Medium	30(32.97)	122(33.61)	152(33.48)
Rich	37(40.66)	114(31.40)	151(33.26)
CBHI status of household			
noninsured	71(78.02)	159(43.80)	230(50.66)
Insured	20(21.98)	204(56.20)	224(49.34)

### Health and health related characteristics of the study population

Regarding the health and health related status of the households, 45(9.91%) of the members of the household had chronic illness/disability while 102 (22.47%) had any acute illness during three months prior to the study. From studied households, 166 (36.56%) had children less than 5 years old. Two hundred ninety (63.88%) households were in good health status [Table 2].

**Table 2:** Health and health related characteristics of household head/spouse by catastrophic health expenditure status, Tehuledere district, Amhara regional state, 2016 [n = 454].

Characteristics	Catastrophic health expenditure		Total
	Yes (%)	No (%)	
Health status of the family(self-reported)			
Poor	14(15.39)	25(6.89)	39(8.59)
Medium	24(26.37)	101(27.82)	125(27.53)
Good	53(58.24)	237(65.29)	290(63.88)
Household having member having chronic illness/disability			
No	74(81.32)	335(92.29)	409(90.09)
Yes	17(18.68)	28(7.71)	45(9.91)
Household having children less than 5 years old			
No	52(57.14)	236(65.01)	288(63.44)
Yes	39(42.86)	127(34.99)	166(36.56)
Household having member greater than 50 years old			
No	52(57.14)	246(67.77)	298(65.64)
Yes	39(42.86)	117(32.23)	156(34.36)
Household member encountered any illness during the past 3 months			
No	60(65.93)	292(80.44)	352(77.53)
Yes	31(34.07)	71(19.56)	102(22.47)

### Catastrophic health expenditure

The level of catastrophic health expenditure among the study population was 91(20%) (95% CI: 16.3, 23.7). Among the households experienced catastrophic health expenditure, 71(15.64%) were noninsured households and 20 (4.41%) were insured. Among households who have not experienced catastrophic health expenditure, 204(44.9%) were insured, whereas the rest 159 (35%) were noninsured households. Monthly average nonfood expenditure was 442.60 ETB (±359.83 ETB). The average monthly health expenditure of the respondents was 44.24 ETB (±102.70 ETB). Of which, 62.06 ETB (±115.65 ETB), and 25.95 ETB (±83.82 ETB) were incurred by noninsured and insured households respectively.

### Factors associated with catastrophic health expenditure

Insurance status, occupation, wealth status, household member with chronic illness/disability and household member encounter any illness during the last three months were significant factors associated with catastrophic health expenditure. Insured households were 81% times less likely to incur catastrophic health expenditure (AOR = 0.19, 95% CI: 0.11–0.34) compared with noninsured households. Households having member with chronic illness/disability were 2.13 times more likely to incur catastrophic health expenditure (AOR = 2.13, 95% CI: 1.01–4.51) compared with those who have no member with chronic illness/disability. Households having member encountered any illness during the past 3 months were 2.44 times more likely to incur catastrophic health expenditure (AOR = 2.44, 95% CI: 1.35–4.40) compared with those who have no member encountered any illness during the past 3 months [Table 3].

**Table 3:** Factors associated with catastrophic health expenditure by the households, Tehuledere district, Amhara regional state, Ethiopia. 2016.

Variables	CHE		Crude OR (95% CI)	Adjusted OR (95% CI)
	Yes	No		
CBHI status				
Uninsured	71	159	1.00	1.00
Insured	20	204	0.22 (0.13,0.38)^***^	0.19 (0.11, 0.34)^***^
Household head/spouse age				
20–29	10	30	1.00	1.00
30–39	23	100	0.70(0.30, 1.61)	0.83(0.33, 2.07)
40–49	22	111	0.60(0.25, 1.40)	0.85(0.33, 2.21)
50–59	10	59	0.51(0.19, 1.36)	0.76(0.26, 2.28)
> = 60	26	63	1.24(0.53, 2.90)	1.73(0.64, 4.68)
Household head/spouse sex				
Male	56	253	1.00	1.00
Female	35	110	1.44(0.89, 2.32)	1.49(0.87, 2.54)
Household head/spouse religion				
Muslim	86	353	1.00	1.00
Orthodox	5	10	2.05(0.68, 6.16)	1.57(0.45, 5.39)
Occupation				
Farming	68	305	1.00	1.00
Non farming	23	58	1.78(1.03, 3.08)^*^	1.96 (1.07, 3.60)^*^
Wealth index of household				
Poor	24	127	1.00	1.00
Medium	30	122	1.30(0.72, 2.35)	1.22(0.64, 2.31)
Rich	37	114	1.72(0.97, 3.05)^*^	1.98(1.07, 3.66)^*^
Health status of household				
Poor	14	25	1.00	1.00
Medium	24	101	0.42(0.19, 0.94)^*^	0.61(0.24, 1.53)
Good	53	237	0.40(0.20, 0.82)^*^	0.82(0.33, 2.05)
Household having member with chronic illness/disability				
No	74	335	1.00	1.00
Yes	17	28	2.75(1.43, 5.28)^*^	2.13(1.01, 4.51)^*^
Household having member with under 5 children				
No	52	236	1.00	1.00
Yes	39	127	1.39(0.87, 2.23)	1.24(0.74, 2.06)
Household having member with above 50 years				
No	52	246	1.00	1.00
Yes	39	127	1.58(0.99, 2.52)	1.22(0.55, 2.73)
Household member encountered any illness during the past 3 months				
No	60	292	1.00	1.00
Yes	31	71	2.13(1.28, 3.52)^**^	2.44(1.35, 4.40)^**^

*p<0.05.

**p<0.01.

***p<0.001.

CHE = Catastrophic health expenditure, OR = odds ratio, CI = confidence interval, CBHI = community based health insurance

### Propensity score matching analysis

This study showed that 20 (4.41%) of insured households and 71(15.64%) of uninsured households had incurred catastrophic health expenditure. Using kernel matching, 224 insured households (treatment group) and 230 noninsured households (control group) matched based on their propensity scores.

Table 4 shows that the average community based insurance effect on insured households was found to be -0.232 points (*t* = -5.94), (95 CI: -0.31_ -0.15) for catastrophic health expenditure. This indicated that Community Based Health Insurance contributed 23.2% reduction to catastrophic health expenditure among insured households [Table 4].

**Table 4:** ATT insured households on catastrophic health expenditure by community, Tehuledre district, Amhara regional state, 2016 [n = 454], [number of replications = 100].

Outcome variable	Treated variable		ATT	SE	t	95% CI
	Insured	Uninsured				
Catastrophic health expenditure	224	228	-0.232	0.039	-5.94	-0.31 _-0.15

**Notes:** The numbers of insured and uninsured households refer to actual kernel matches; ATT estimation with kernel matching method. ATT–average treatment of treated; SE-standard error; CI-confidence interval.

## Discussion

This study aimed to assess the effect of community based health insurance on catastrophic health expenditure in the rural community of northeast Ethiopia. We also identified the predictors of catastrophic health expenditure.

Our study revealed that 20% of the households faced catastrophic health expenditure. This finding suggests that significant proportion of informal sector households’ face catastrophic health expenditure which is an obstacle for basic health service access and universal health coverage. Studies conducted in Rwanda and Tanzania reported that 20.1% and 18% of the households had catastrophic health expenditure respectively [24, 25]. Similarly, the number of households categorized as having catastrophic health expenditure in Kenya was 18% at 30% threshold to 22.2% at 10% threshold (4.05%) [26]. However, this finding is much higher than studies done in Iran [27, 28]. The possible explanation for the observed difference might be due to socio-economic difference between the households of Iran and Ethiopia.

The multivariable logistic regression revealed that CBHI membership, occupation, wealth status, chronic illness and acute illness were statistically and significantly associated with catastrophic health expenditure of the households. Insured households were 81% times less likely to incur catastrophic health expenditure compared with the uninsured households. Similarly, the propensity score matching model showed that CBHI membership decreased catastrophic health expenditure by 23.2% (ATT = -0.232; t = -5.94; 95% CI,-0.31_-0.15). This finding is supported by a study done in Rwanda, in which being insured household decreased catastrophic health expenditure by 15.1% (ATT = -0.151) [24]. Similar studies found that insured household has less catastrophic health expenditure compared with the noninsured households [12, 29]. However, studies conducted in china, India, Laos and Vietnam found that CBHI membership has no effect on CHE (15, 16, 18–21). The possible explanation could be due to the difference in the implementation approach of the health insurance scheme. For instance, the national health insurance scheme of India only covers hospitalization while the predominant share in household out of pocket expenses are accounted for by outpatient episodes, whereas the community based health insurance in Ethiopia coverers both outpatient and inpatient service utilization at public health facilities. Similarly, high dropout rate was reported in India.

The odds of incurring CHE among non-farming households (merchants and laborers) were 1.96 times more likely than farming households. This indicates the relatively high out of pocket health expenditure incurred by the non-farming households. This study revealed that rich households were 1.98 times more likely to incur catastrophic health expenditure compared with poor households. This finding is supported by studies done in Nigeria, Egypt and Cambodia. However, this finding is not consistent with other studies conducted in Iran [27, 28], Tanzania [25] and North India [30].

The possible explanation is that the poor households may seek low quality health care or avoid seeking health care at all due to their inability to pay for health services. On the hand, the better off households may have been by-passing the public health facilities to get better quality health care at the private hospitals in which they are expected to pay more than the public hospitals for the same services. In addition, the government of Ethiopia provides fee waiver for the poorest households to get the health care in public health facilities. So, the rich households may incur more out of pocket payment than their counterparts.

This study revealed that households having a member with chronic illness/disability were 2.13 times more likely to incur catastrophic health expenditure compared with those who have no member with chronic illness/disability. This finding was in line with other studies done in Tanzania [25] and China [18, 19]. However, this finding was not supported by a study done in Burkina Faso [31] in which having a disabled member in the household has no effect on CHE. The possible explanation could be the difference in threshold/cut-off value taken to determine catastrophic health expenses. This study found that households having member encountered any illness during the past 3 months were 2.44 times more likely to incur catastrophic health expenditure compared with those who have no member encountered any illness during the past 3 months. This finding indicates that households having member encountered any illness are at higher risk for catastrophic health expenditure. This might be due to they are supposed to incur out of pocket payment in order to get health care services.

This study has some limitations. First, there might be social desirability bias because information on income and expenditure relied on the report of the heads of household/spouse. Second, the severe drought happened during the study period may have affected the results of the study because of fee waiver and subsided supply of materials. Third, propensity score ignores the effects of unobserved characteristics which may have an effect on the results of the study. Therefore, the findings of this study should be interpreted taking these in to consideration. Despite those limitations, this study provides useful insights about the level of catastrophic health expenditure and the effect of community based health insurance on catastrophic health expenditure in northeast Ethiopia and this study should serve as a base for more detailed investigation in Ethiopia.

## Conclusion

The present study showed that the level of catastrophic health expenditure in our study was high. Our study also showed that community based health insurance has significant financial protection for catastrophic health expenditure in northeast Ethiopia. CBHI status, occupation, wealth status, household with chronic illness/disability member and household member encountered illness during the last 3 months were the factors statistically and significantly associated with catastrophic health expenditure. Hence, the Ethiopian health insurance agency need to improve the CBHI benefit packages to prevent insured households from passing government health facilities. In addition, Amhara regional health bureau needs to develop supportive strategies for households who had a member with chronic illness/disabilities, considering fee waiver for the poor non-farming households and improve the quality of health services in government owned health facilities to benefit both poor and rich households. Tehuledere district CBHI coordinating office needs to scale up the community-based health insurance to nonmember households so as to prevent financial burdens. Besides, creating awareness and community mobilization on the importance of community based health insurance is very important.

## Supporting information

S1 File Amharic and English version questionnaire used to collect data in Northeastern Ethiopia. (DOCX)
