# Supplementary material for: The effect of community based health insurance on catastrophic health expenditure in Northeast Ethiopia: A cross sectional study
Source: PLoS One. 2018 Oct 18;13(10):e0205972. doi: 10.1371/journal.pone.0205972 (PMC6193712; doi:10.1371/journal.pone.0205972)
Supplement: S1 File — (DOCX) [file pone.0205972.s001.docx]

**Annex1: Amharic version Questionnaire**

ቃለ መጠይቅ፤ በሰሜን ምስራቅ ኢትዮጵያ አማራ ክልላዊ መንግስት ተሁለደሬ ወረዳ የማህበረሰብ አቀፍ የጤና መድህን በያልተጠበቀ የጤና ወጪ የሚያመጣው ውጤት የሚደረግ ጥናት 2008.

የተመረጠው ቤተሰብ መለያ

1. ቀበሌ _____________________
2. ጎጥ/መንደር_____________________
3. የቤተሰቡ መለያ ቁጥር______________________
4. ቤተሰቡ የማዐጤመ አባል ነው? 1. አዎ 2. አይደለም
5. ቤተሰቡ የጤና መድህን አባል የሆነበት ጊዜ____________________
6. የቃለ መጠየቅ ቁጥር _______________________

መመሪያ

1. የሚሰጥህን/ሽን መልስ በማክበብ አሳዩ
2. በመጀመሪያ የቤተሰቡን መሪ ይጠይቁ፡ ከለለ/ከለለች ሚስቱን/ባሏን ይጠይቁ፡፡

| ክፍል 1. የቤተሰቡን ማህበራዊ አኗኗር የሚገልፅ መጠይቅ | | | |
| --- | --- | --- | --- |
| ጥ.ቁ. | ጥያቄዎች | መልስ | ምርመራ |
| 101 | የቤተሰቡ መሪ/ባለቤት እድሜ ስንት ይሆናል? | _______________ |  |
| 102 | የቤተሰቡ መሪ/ባለቤት ጾታ | 1. ወንድ 2. ሴት |  |
| 103 | የቤተሰቡመሪ/ባለቤት አግብተዋል? | 1. ያላገቡ  2. ያገቡ  3. ተፋተዋል  4. ባለቤቱ የሞተችበት/ባለቤቷ የሞተባት  5. ሌላ |  |
| 104 | የቤተሰቡ መሪ/ባለቤት የምን ሀይማኖት ተከታይ ነው/ናት? | 1. ኦርቶዶክስ  2. ሙስሊም  3. ፕሮቴስታንት  4. ሌሎች, ግለጽ--------------- |  |
| 105 | የቤተሰቡ የአባላት ቁጥር ስንት ነው? | _______________________ |  |
| ክፍል 2. የቤተሰቡ ማህበራዊ ኢኮኖሚ ሁኔታ የሚገልፅ መጠይቅ | | | |
| 106 | የቤተሰቡ መሪ/ባለቤት የትምህርት ደረጃ ምን ያህል ነው? | 1. ማንበብ እና መጻፍ የማልችል 2. ት/ቤት ገብቼ የማላውቅ ግን ማንበብም መፃፍም የምችል 3. የመጀመሪያ ደረጃ (1-8) 4. የሁለተኛ ደረጃ (9-12) 5. የከፍተኛደረጃ(ከ12 በላይ) |  |
| 107 | የቤተሰቡ መሪ/ባለቤት ስራ ምንድን ነው? | 1. ግብርና  2. ነጋዴ  3. የቀን ሰራተኛ  4. የቤት እመቤት  5. ሌሎች, ግለጽ__________ |  |
| ክፍል 3. የጤና እና ጤና ነክ ሁኔታዎች መጠይቅ | | | |
| 108 | የቤተሰቡን የጤና ሁኔታ እንዴት ታየዋልህ/ሽ? | 1. በጣም ዝቅተኛ 2. ዝቅተኛ 3. መካከለኛ 4. ጥሩ 5. በጣም ጥሩ |  |
| 109 | አንተ ወይም ከቤተሰቡ አባል ስር የሰደደ በሽታ/ጉድለት ያለበት አለ? | 1. አዎ 2. የለም |  |
| 110 | በቤተሰቡ ውስጥ እድሜያቸው ከ5 አመት በታች የሆኑ ህፃናት አሉ? | 1. አዎ 2. የለም |  |
| 111 | በቤተሰቡ ውስጥ እድሜያቸው ከ50 አመት በላይ የሆኑ አሉ? | 1. አለ 2. የለም |  |
| 112 | በባለፉት ሶስት ወራት ከቤተሰቡ አባል የታመመ ነበር? | 1. አዎ 2. የለም |  |

| ክፍል 4. የሃብት ጠቋሚ ጥያቄዎች | | | | |
| --- | --- | --- | --- | --- |
| ጥ.ቁ. | ጥያቄወች | መልስ | ብዛት | ም. |
| 113 | ከእነዚህ እንሰሳት ውስጥ ቤተሰቡ ምን ያህል አለው? | 1. በሬዎች፣ላሞች፣ኮርማዎች, |  |  |
|  |  | 1. ጥጃ፣ወይፈን፣ጊደር |  |  |
|  |  | 1. ፍየል |  |  |
|  |  | 1. በግ |  |  |
|  |  | 1. አህያ |  |  |
|  |  | 1. ፈረስ |  |  |
|  |  | 1. በቅሎ |  |  |
|  |  | 1. ግመል |  |  |
|  |  | 1. የንብ ቀፎዎች |  |  |
|  |  | 1. ዶሮ |  |  |
|  |  | 1. ሌሎች‹ግለጽ ------- |  |  |
| 114 | ቤተሰቡ አለው? | 1. ዘመናዊ አልጋ | 1. አወ2.የለም |  |
|  |  | 1. ሬድዮ/ቲቭ የሚሰራ | 1. አወ 2.የለም |  |
|  |  | 1. ሞባይል | 1. አዎ2.የለም |  |
|  |  | 1. የውሀ መሳቢያ | 1. አዎ2.የለም |  |
|  |  | 1. የጥጥ/ስፖንጅ/ስፐሪንግ/ፍራሽ | 1. አዎ2.የለም |  |
| 115 | ቤተሰቡ ምን አይነት ሽንት ቤት አለው? | 1. ምንም 2. የተሻሻለ ሽታ አልባ የጉድጓድ መጸዳጃ ቤት 3. ባህላዊ የጉድጓድ መጸዳጃ ቤት 4. ሌሎች፣ግለፅ------- |  |  |
| 116 | የቤቱጣሪያምአይነትነው? | 1. ቆርቆሮ 2. ሳር 3. ሌሎች፣ ግለጽ |  |  |
| 117 | የመኖሪያ ቤቱ ግድግዳ ምን አይነት ነው? | 1. ከእንጨት የተሰራ 2. ጭቃ 3. ሌሎች ግለጽ |  |  |
| 118 | ማዕድ ቤት አለ? | 1.አዎ  2. የለም |  |  |
| 119 | ለእንሰሳት ማደሪያ የተለየ ቤት አለዎት? | 1. አዎ 2. የለም |  |  |
| 120 | የቤተሰቡ የመሬት ይዞታ በሄክታር ምን ያህል ነው? | _______________በሄክታር |  |  |
| 121 | ከነዚህ ምርቶች ውስጥ በግምት በባለፈው 1 አመት ቤተሰቡ ምን ያህል አምርቶ ሸጧል? | 1. ጤፍ (ብር) |  |  |
|  |  | 1. ማሽላ (ብር) |  |  |
|  |  | 1. በቆሎ (ብር) |  |  |
|  |  | 1. ስንዴ (ብር) |  |  |
|  |  | 1. ባቄላ (ብር) |  |  |
|  |  | 1. አተር (ብር) |  |  |
|  |  | 1. ሽምብራ (ብር) |  |  |
|  |  | 1. ምስር (ብር) |  |  |
|  |  | 1. ጫት (ብር) |  |  |
|  |  | 1. ቅጠላ ቅጠል/ፍራ ፍሬ (ብር) |  |  |
|  |  | 1. ሌሎች ጥቀስ |  |  |

| ክፍል 5. ምግብ ነክ ያልሆኑ ወጪዎች: የባለፉት 12 ወሮች ጠቅላላ ወጪ በብር.  ላልተገዛ ዝርዝር ዜሮ ሙላ፤ | | | | | | | |
| --- | --- | --- | --- | --- | --- | --- | --- |
|  | 122 | 123 | 124 |  | 122 | 123 | 124 |
|  | የወጪ አይነት | ገዝተሀል? | ወጪ |  | የወጪ አይነት | ገዝተሀል? | ወጪ |
|  |  | 1.አዎ  2.የለም | ብር |  |  | 1.አዎ  2.የለም | ብር |
| 1 | የልብስና ተያያዥ ጉዳዮች ወጪ |  |  | 3.8 | ለበሬ ክራይ |  |  |
| 1.1 | ልብሶች/ጫማዎች/ጨርቅ ለወንዶች |  |  | 3.9 | ካሳ፣ቅጣት፣ብድር |  |  |
| 1.2 | ልብሶች/ጫማዎች/ጨርቅ ለሴቶች |  |  | 3.10 | ሌሎች |  |  |
| 1.3 | ልብሶች/ጫማዎች/ጨርቅ ለወንድ ልጆች |  |  | 4. | የጤና ወጪ |  |  |
| 1.4 | ልብሶች/ጫማዎች/ጨርቅ ለሴት ልጆች |  |  | 4.1 | ዘመናዊ ህክምናና መድሀኒት |  |  |
| 1.5 | ልብሶች/ጫማዎች/ጨርቅ ለህጻናት |  |  | 4.1.1 | መድሃኒቶች |  |  |
| 1.6 | የአልጋ ልብስ(አንሶላ፣ፎጣ፣ብርድ ልብስ) |  |  | 4.1.2 | ክፍያ |  |  |
| 1.7 | የውበት መዋቢያ(ቅቤ፣የጸጉር ቅባት፣ሽቶ …) |  |  | 4.1.3 | መጓጓዣ |  |  |
| 1.8 | ሌሎች |  |  | 4.1.4 | ለምግብና መጠለያ |  |  |
| 2. | የቤትና ተያያዥ ጉዳዮች ወጪ |  |  | 4.15 | ሌሎች |  |  |
| 2.1 | የማእድ ቤት እቃዎች(ድስት…..) |  |  | 4.2 | የባህላዊ ህክምና  መድሀኒት |  |  |
| 2.2 | የቤት እቃ |  |  | 4.2.1 | መድሀኒቶች |  |  |
| 2.3 | ፋኖስ/የእጅ ባትሪ |  |  | 4.2.2 | ክፍያ |  |  |
| 2.4 | ወንፊት፣ሰፌድ፣መሶብ |  |  | 4.2.3 | መጓጓዣ |  |  |
| 2.5 | ማጓጓዣ (ከጤናና ምርት ውጪ) |  |  | 4.2.4 | ምግብና መጠለያ |  |  |
| 2.6 | የግንባታ ዕቃዎች |  |  | 4.2.5 | ሌሎች |  |  |
| 2.7 | ለጥገና |  |  | 5 | የትምህርት ወጪ |  |  |
| 2.8 | ክብሪት |  |  | 5.1 | የትምህርት ቤት (ክፍያ) |  |  |
| 2.9 | ባትሪ ድንጋይ |  |  | 5.2 | ሌሎች |  |  |
| 2.10 | ጧፍና አጣን |  |  | 6 | የግብርና ግብአትና እንሰሳት ወጪ |  |  |
| 3.11 | ሳሙናና ኦሞ |  |  | 6.1 | ማዳበሪያ |  |  |
| 3.12 | ሌሎች |  |  | 6.2 | ፀረ ተባይና ፀረ አረም |  |  |
| 3.13 | ሌሎች |  |  | 6.3 | ዘርና ችግኝ |  |  |
| 3 | የማህበራዊ ጉዳይ ወጪ |  |  | 6.4 | የሰብል ምርት ሰራተኛ |  |  |
| 3.1 | ለክብረ በዓል(ለቀለበት፣ጥሎሽ፣. |  |  | 6.5 | ለሰብል ምርትና ሽያጪ ማጓጓዣ |  |  |
| 3.2 | የእድር ክፍያ |  |  | 6.6 | ለከብት እረኛ |  |  |
| 3.3 | ለቤተ ክርስቲያን የተሰጠ ምፅዋት |  |  | 6.7 | ለእንሰሳት መኖ |  |  |
| 3.4 | ግብርና ቀረጥ |  |  | 6.8 | ለእንሰሳት ህክምና |  |  |
| 3.5 | በጎ አድራጎት (እርባን) |  |  | 7 | ከሞት ጋር የተያያዙ ወጪዎች |  |  |
| 3.6 | ክራይ(ከበሬና መሬት ውጪ) |  |  | 7.1 | ለቀብር፡ ተዝካር፡ሰደቃ |  |  |
| 3.7 | የመሬት ክራይ |  |  | 7.2 | ሌሎች |  |  |
| ጠቅላላ ድምር | |  |  |  |  |  | |

ቃለ ምልልሱ አልቋል አመሰግናለሁ

ይህን ቃለ ምልልስ ያደረገው ሰው ስምና ፊርማ ________________________

የቅርብ ክትትል ያደረገው ሰው ስምና ፊርማ ___________________________

**Annex1: English version Questionnaire**

**Questionnaire on Effect of Community Based Health Insurance on Catastrophic Health Expenditure in Tehuledere District Amhara Regional State, North East Ethiopia, 2016**

**Identification of the selected household**

1. Kebele _____________________
2. Got/village___________________
3. Household family No.____________________
4. Household participation in CBHI 1. Yes 2. No
5. Time being member to CBHI ___________________
6. Questionnaire number___________________

**INSTRUCTION:**

1. Circle the response
2. First ask the household head, if not present ask the spouse.

| **PART I. HOUSEHOLD SOCIO DEMOGRAPHIC FACTORS** | | | |
| --- | --- | --- | --- |
| Q.NO | Questions | Response | Remark |
| 101 | Age of the household head/spouse | _______________ |  |
| 102 | Sex of the household head/spouse | 1. Male 2. Female |  |
| 103 | Marital status of the household head/spouse | 1. single  2. Married  3. Divorced  4. Widowed  5. others |  |
| 104 | Religion of the household head/spouse | 1. Orthodox  2. Muslim  3. Protestant  4. others, specify---------------- |  |
| 105 | What is the size of family of the household? | ___ |  |
| **PART II. HOUSEHOLD SOCIO ECONOMIC FACTORS** | | | |
| 106 | Educational status of the household head/spouse | 1. Illiterate 2. Reading and writing 3. Primary education( 1-8) 4. Secondary education (9-12) 5. Tertiary education (above 12) |  |
| 107 | Occupation/employment of the household head/spouse | 1. farming  2. merchant  3. laborer  4. housewife  5. Other, specify__________ |  |
| **PART III. HEALTH AND HEALTH RELATED FACTORS** | | | |
| 108 | How do you rate the health status of your family? | 1. Very poor 2. Poor 3. Medium 4. Good 5. Very good |  |
| 109 | Do you or other member of the household have chronic illness and/or disability? | 1. Yes 2. No |  |
| 110 | Are there under 5 children in the household? | 1. Yes 2. No |  |
| 111 | Are there above 50 household members? | 1. Yes 2. No |  |
| 112 | Have any member of the family encountered any illness during the past 3 months? | 1. Yes 2. No |  |

| **PART IV. WEALTH INDEX QUESTIONS** | | | | | | |
| --- | --- | --- | --- | --- | --- | --- |
| Q.No | Questions | Response | Numbers | | | Skip |
| 113 | How many of these animals do this household own? | 1. Oxen, cows, bulls | |  | |  |
|  |  | 1. Calf and heifer | |  | |  |
|  |  | 1. Goat | |  | |  |
|  |  | 1. Sheep | |  | |  |
|  |  | 1. Donkey | |  | |  |
|  |  | 1. Horse | |  | |  |
|  |  | 1. Mule | |  | |  |
|  |  | 1. Camel | |  | |  |
|  |  | 1. Beehives | |  | |  |
|  |  | 1. Chicken | |  | |  |
|  |  | 1. Other, specify _____ | |  | |  |
| 114 | Does your household have? | 1. Modern bed | | 1. Yes 2. No | |  |
|  |  | 1. Radio/TV functional | | 1. Yes 2. No | |  |
|  |  | 1. Mobile | | 1. Yes 2. No | |  |
|  |  | 1. Water pump | | 1. Yes 2. No | |  |
|  |  | 1. Cotton/sponge/spring mattress | | 1. Yes 2. No | |  |
| 115 | What kind of latrine does your family have? | 1. None 2. Ventilated improved latrine 3. Traditional latrine 4. Others, specify____ | |  | |  |
| 116 | What is the type of roof of the house? | 1. Corrugated sheet 2. Thatch roof 3. Others specify | |  | |  |
| 117 | What is the wall of your residence house made of? | 1. Wooden structure 2. Mud 3. Others, specify_____ | | |  |  |
| 118 | Do you have kitchen? | 1.Yes  2. No | | |  |  |
| 119 | Do you have separate rooms for cattle? | 1. Yes 2. No | | |  |  |
| 120 | What is the total farm size holding of the household in Hectares? | Size in ____________hectares | | |  |  |
| 121 | Approximately, how much of these products did your household produced and sold during the last 1 year? | 1. Teff in (Birr) | | |  |  |
|  |  | 1. Sorghum in (Birr) | | |  |  |
|  |  | 1. Maize in( Birr) | | |  |  |
|  |  | 1. Wheat in (Birr) | | |  |  |
|  |  | 1. Bean in (Birr) | | |  |  |
|  |  | 1. Pea in (Birr) | | |  |  |
|  |  | 1. Chickpeas in (Birr) | | |  |  |
|  |  | 1. Lentils in (Birr) | | |  |  |
|  |  | 1. Khat in (Birr) | | |  |  |
|  |  | 1. Vegetables/fruits in (birr) | | |  |  |
|  |  | 1. Others, specify______ | | |  |  |

| **PART V. NON-FOOD EXPENDITURE: THE LAST 12 MONTH TOTAL EXPENDITURE IN BIRR. FOR NON PURCHASED ITEM FILL ZERO** | | | | | | | |
| --- | --- | --- | --- | --- | --- | --- | --- |
|  | 122 | 123 | 124 |  | 122 | 123 | 124 |
|  | **Types of expenditure** | **Did you purchase?** | **Expense** |  | **Types of expenditure** | **Did you purchase?** | **Expense** |
|  |  | 1.yes  2.No | Birr |  |  | 1.yes  2.No | Birr |
| 1 | **Expenditure on clothes and related** |  |  | 3.9 | Rent for oxen |  |  |
| 1.1 | Clothes/shoes/fabric for men |  |  | 3.10 | Compensation, penalty and credit |  |  |
| 1.2 | Clothes/shoes/fabric for women |  |  | 3.11 | others |  |  |
| 1.3 | Clothes/shoes/fabric for boys |  |  | 4. | **Expenditure on health** |  |  |
| 1.4 | Clothes/shoes/fabric for girls |  |  | 4.1 | Modern medical treatment and medicines |  |  |
| 1.5 | Cloth/shoes/fabric for babies |  |  | 4.1.1 | Drugs |  |  |
| 1.6 | Linens (sheets, towels, blankets) |  |  | 4.1.2 | Fees |  |  |
| 1.7 | Cosmetics (hair oil, butter, perfume) |  |  | 4.1.3 | Transportation |  |  |
| 1.8 | Others |  |  | 4.1.4 | Lodging/board |  |  |
| 2.0 | **Expenditure on housing and related** |  |  | 4.15 | Others |  |  |
| 2.1 | Kitchen equipment (cooking pots, etc.) |  |  | 4.2 | Traditional medicine and healers |  |  |
| 2.2 | Furniture |  |  | 4.2.1 | Drugs |  |  |
| 2.3 | Lamp/torch |  |  | 4.2.2 | Fees |  |  |
| 2.4 | Sieve (wonfiet), sefed, mesob, etc |  |  | 4.2.3 | Transportation |  |  |
| 2.5 | Transport (other than health purpose, crop production and crop sale) |  |  | 4.2.4 | Lodging/board |  |  |
| 2.6 | Building materials |  |  | 4.2.5 | Others |  |  |
| 2.7 | Repair and maintenance |  |  | 5 | **Expenditure on education** |  |  |
| 2.8 | Matches |  |  | 5.1 | Education (school fees) |  |  |
| 2.9 | Batteries |  |  | 5.2 | Others |  |  |
| 2.10 | Candles ,incense |  |  | 6 | **Expenditure on agricultural**  **Inputs and livestocks** |  |  |
| 2.11 | Soap/OMO |  |  | 6.1 | Fertilizer |  |  |
| 2.12 | Others |  |  | 6.2 | Pesticides (incl. fungicides and herbicides) |  |  |
| 2.13 | Others |  |  | 6.3 | Seeds and young plants |  |  |
| 3 | **Expenditure on social obligations** |  |  | 6.4 | Labor for crop production |  |  |
| 3.1 | Ceremonial expenses (gold, dowry for spouse) |  |  | 6.5 | Transport related to crop production and crop sale |  |  |
| 3.2 | Contributions to *iddir* |  |  | 6.6 | Labor for herding |  |  |
| 3.3 | Donations to the church |  |  | 6.7 | Animal feed |  |  |
| 3.4 | Taxes and levies |  |  | 6.8 | Veterinary services/medicines |  |  |
| 3.5 | Compensation and penalty |  |  | 6.9 | other expenses |  |  |
| 3.6 | Voluntary contributions (including ereban) |  |  | 7 | **Death related expenditure** |  |  |
| 3.7 | Rent (other than oxen and land |  |  | 7.1 | Funerals,*Teskar* and *sedeka* |  |  |
| 3.8 | Rent for land |  |  | 7.2 | others |  |  |
| **Grand total** | |  |  |  |  |  | |

The interview is ended. Thank you.

Interviewer’s name _________________signature _________date________________

Supervisor name ___________________ signature ________ date___________
